# Mixed depression and suicidality in oncology outpatients

**DOI:** 10.1192/j.eurpsy.2023.784

**Published:** 2023-07-19

**Authors:** E. Bernardi, A. Catinari, O. M. Ferrara, G. Bartolucci, M. Ciliberto, G. Carriero, L. Di Benedetto, E. M. Marzo, G. Donofrio, S. Ruggiero, G. Sani, A. Simonetti

**Affiliations:** 1 Neuroscience, Section of Psychiatry, Fondazione Policlinico Universitario Agostino Gemelli IRCCS; 2 Neuroscience, Section of Psychiatry, Università Cattolica del Sacro Cuore, Rome, Italy; 3 Menninger Department of Psychiatry and Behavioral Sciences, Baylor College of Medicine, Houston, TX, United States

## Abstract

**Introduction:**

Mixed depression (MxD), is a nosologic entity characterized by the presence of excitatory symptoms during a depressive episode. MxD embeds high levels of chronicity, functional impairment and suicidality. The assessment of MxD in a subpopulation that features high levels of fragility, such as oncology patients, represent a pivotal strategy to reduce illness burden and suicidality in these subjects

**Objectives:**

The aim of the present project is to assess the characteristics of MxD in oncology outpatients and to compare them with those of outpatients without oncological comorbidity.

**Methods:**

Forty-two oncology outpatients with MxD (ONC-MxD); 34 oncology outpatients and inhibited depression (ONC-inhib); 187 outpatients with MxD without oncological comorbidity (MxD); 224 outpatients with inhibited depression without oncological comorbidity (Inhib) and 168 healthy controls (HC) have been recruited. Analyses made include comparisons of demographic and clinical variables, depression severity, excitatory symptoms, suicidality and functional impairment.

**Results:**

Oncology outpatients with depressive disorder showed greater severity of depressive symptoms and greater functional impairment than those without oncological comorbidity (F=187.08; *p*<.001; F=54.08; *p*<.001, respectively). ONC-inhib showed greater inhibition than Inhib (*p*<.001), whereas no differences in levels of excitatory symptoms are present between MxD e ONC-MxD (*p*=.159). ONC-DMX have a more recent diagnosis of cancer than ONC-inib (F=13.39, *p*<.001) and higher rates of suicidal ideation (χ²=11.89; *p*=.008).

**Conclusions:**

Cancer might worsen depression severity, especially in its inhibitory component. Relationships between onset of cancer, excitatory symptoms and suicidality suggest that the period following the diagnosis of cancer is the one at higher risk for suicide. Strategies aiming to treat excitatory symptoms in such period might help reduce risk of suicide in oncology patients.

**Disclosure of Interest:**

None Declared

